# Bile Acid Sequestration Attenuates *Desulfovibrio*-Induced Hepatic Injury

**DOI:** 10.3390/microorganisms14010079

**Published:** 2025-12-30

**Authors:** Songfan Yang, Lingxi Zhou, Jie Dong, Sifan Wang, Yuzheng Xue, Yilin Ren, Yan Geng

**Affiliations:** 1 School of Life Sciences and Health Engineering, Department of Gastroenterology, Affiliated Hospital of Jiangnan University, Jiangnan University, Wuxi 214122, Chinayuzhengxue_ahjnu@126.com (Y.X.); 2 The Key Laboratory of Industrial Biotechnology, Ministry of Education, School of Biotechnology, Jiangnan University, Wuxi 214122, China; 7200201035@stu.jiangnan.edu.cn; 3 Department of Microbiology, Tumor and Cell Biology, Centre for Translational Microbiome Research (CTMR), Karolinska Institute, 17177 Stockholm, Sweden

**Keywords:** *Desulfovibrio*, bile acids, gut microbiota, liver injury, enterohepatic circulation

## Abstract

*Desulfovibrio* (DSV), sulfate-reducing gut bacteria that generate hydrogen sulfide (H_2_S), can impact host health through diverse mechanisms including bile acid (BA) metabolism. Although intestinal overgrowth of DSV expands the BA pool and promotes liver injury, its causal role in hepatic pathophysiology remains incompletely defined. Here, by employing complementary interventions of cholic acid (CA) supplementation and the BA sequestrant cholestyramine in mouse models, we show that DSV-driven liver injury is mediated by aberrant BA metabolism coupled with gut microbial remodeling. CA alone induced overt hepatic damage, whereas supplemental DSV did not further exacerbate injury caused by excessive CA. Intervention with the BA sequestrant cholestyramine markedly attenuated DSV-elicited hepatic inflammatory and histological alterations, which were associated with an upregulation of the intestinal BAs pool. Hepatic expression of BAs synthetic genes *Cyp7a1* and *Cyp8b1* verified a negative-feedback regulation of BA metabolism upon treatments. 16S rRNA gene sequencing revealed that CA, DSV, and the cholestyramine all significantly influenced the gut microbiota. CA reduced microbial richness and drove community separation, while DSV intervention under high BA conditions enriched specific biomarkers including *Eubacterium ventriosum* and *Enterorhabdus*. Notably, the administration of cholestyramine attenuated these DSV-associated microbial shifts and further reduced overall species richness, confirming the integral role of BA dynamics in shaping the gut microbial community. Collectively, our research reveals the intricate link between DSV, BAs, and gut microbiota in liver injury, and suggests that modulation of BAs may hold therapeutic potential for DSV-associated liver hepatic conditions.

## 1. Introduction

As the primary functional components of bile, bile acids (BAs) are produced within liver cells from cholesterol, stored in the gallbladder, and later discharged into the digestive tract [1,2]. Beyond their digestive role, BAs function as key signaling molecules in metabolic regulation [3,4]. Approximately 95% of BAs are reabsorbed in the ileum and returned to the liver via the portal vein, completing the enterohepatic circulation—a process central to maintaining BA pool homeostasis [5,6]. Disruption to this homeostasis, for example when BA levels or composition become abnormal, can induce liver damage. Clinical studies have shown that BA metabolic abnormalities are present in diseases such as non-alcoholic fatty liver disease, cholestatic liver injury, and liver fibrosis [7,8,9]. Notably, the use of BA chelating agents can effectively alleviate liver injury caused by the abnormal accumulation of BAs, which further corroborates the critical role of BA homeostasis in liver health [10,11].

The gut microbiota and BAs form a two-way regulatory loop. Microbial enzymes transform host BAs, resetting both the size and composition of the intestinal BA pool. These modified BAs then act as selective chemical cues, inhibiting or promoting specific taxa and gradually reshaping microbial community structure [12,13,14]. When this balance is disrupted, dysbiosis of the gut microbiota can further interfere with BA synthesis and metabolism, leading to liver damage and exacerbating disease progression [15,16,17].

*Desulfovibrio* (DSV) is a common type of sulfate-reducing bacterium characterized by its core metabolic function of utilizing sulfate as an electron acceptor to produce hydrogen sulfide (H_2_S) [18]. Under normal physiological conditions, DSV is present in low numbers in the gut and maintains a stable symbiotic relationship with the host. However, numerous studies have reported a positive correlation between its abundance and various diseases [19,20,21]. In addition, DSV can promote the formation of gallstones by affecting BA metabolism. It interferes with the FXR/CYP7A1 signaling pathway in the liver, altering the composition of the BA pool and ultimately leading to cholesterol supersaturation in bile. This contributes to the formation of gallstones [22]. Our previous studies showed that exogenous administration of *Desulfovibrio desulfuricans* ATCC 29577 to mice induced hepatic inflammation and elevated BA levels [23]. However, whether DSV perturbs BAs to elicit hepatic injury, and whether intervening in BA levels can alleviate this injury, remain to be determined.

In this study, we hypothesized that DSV-induced liver injury is dependent on the expansion of the intestinal BA pool and that reducing intestinal BA levels would ameliorate this injury. We aimed to investigate the role of BA homeostasis in DSV-induced liver injury by employing two contrasting interventions in mouse models, cholic acid supplementation to induce BA excess and the BA sequestrant cholestyramine to deplete intestinal BAs. Additionally, we analyzed the gut microbiota to explore how the interaction between gut microbiota and BAs contributes to DSV-induced liver injury.

## 2. Materials and Methods

### 2.1. Bacterial Strain and Culture Conditions

*D. desulfuricans* (ATCC #29577 = CGMCC 1.5189) was purchased from the China General Microbiological Culture Collection Center (Beijing, China). It was incubated anaerobically at 37 °C in an anaerobic chamber (<0.1% O_2_) in a routine culture medium EDCM containing the following: 0.5 g/L potassium dihydrogen phosphate, 1.0 g/L dipotassium hydrogen phosphate, 1.0 g/L sodium sulfate, 0.06 g/L calcium chloride, 0.06 g/L magnesium sulfate heptahydrate, 1.0 g/L yeast extract, 3.5 g/L sodium lactate, and 0.5 g/L ferrous sulfate. To maintain stability, sodium sulfate, magnesium sulfate heptahydrate, and calcium chloride were pre-sterilized by filtration (0.22 μm) and added after the autoclaving and mixing steps of the base medium. The final pH was calibrated to 7.4 ± 0.2. Prior to animal gavage, bacterial viability was confirmed by plating serial dilutions on EDCM agar plates and incubating anaerobically at 37 °C for 48–72 h. The optical density at 600 nm (OD_600_) of liquid cultures was measured and correlated with colony-forming unit (CFU) counts from plate assays to ensure accurate and consistent dosing.

### 2.2. Animals and Experimental Designs

Male BALB/c mice were obtained from Shanghai Slac Laboratory Animal Ltd. (Shanghai, China) and housed at Jiangnan University, with the approval number JN.No. 20230615b0801006[302]. Mice were housed under specific-pathogen-free (SPF) conditions with a 12-h light-dark cycle, 40 ± 5% humidity, and a temperature of 20–22 °C, and had free access to a regular diet (Cat: M01-F, Shanghai Puteng Biotechnology Co., Ltd., Shanghai, China) and water. The principal nutritional composition (on a dry matter basis) of the diet was approximately 18% protein, 7% fat, 64% carbohydrates, and less than 5% crude fiber. After a one-week acclimatization period, they were randomly assigned to experimental groups (n = 5 per group) for subsequent treatments.

For mice in the cholic acid (CA) treatment groups, the grouping and treatments were as follows: The CTRL group with no intervention received only a regular diet (Cat:1010063, Jiangsu Collaborative Medical Biotechnology Co., Nanjing, China). The CA group was fed a regular diet containing 0.4% cholic acid. The DSV-CA group received a regular diet containing 0.4% cholic acid and was administered DSV via gavage three times a week. Each gavage dose consisted of 2 × 10^8^ CFU suspended in 200 μL PBS, a regimen based on our previous study in which this dose effectively induced relevant hepatic phenotypic alterations [23]. During the gavage period for the third group, mice in the first and second groups were also administered 200 μL PBS via gavage. The experimental period for this group was 28 days.

For mice in the cholestyramine treatment groups, the grouping and treatments were as follows: The CTRL group with no intervention received only a regular diet. The DSV group was fed a regular diet and administered DSV via gavage three times a week, with each dose consisting of 2 × 10^8^ CFU suspended in 200 μL PBS. The DSV-C group was administered DSV via gavage three times a week and fed a regular diet containing 2% cholestyramine. In contrast to the second and third groups, which underwent gavage, mice in the first group received 200 μL PBS via gavage. The experimental period for this group was 28 days.

### 2.3. Pathology Analysis

After fixation in 4% paraformaldehyde, liver tissues were dehydrated, embedded in paraffin, and sliced into 4 μm-thick sections for morphological examination. Sections underwent Hematoxylin and Eosin (H&E) staining. Antigen retrieval was performed using citrate buffer. Then, sections were subjected to Adhesion G protein-coupled receptor E1 (F4/80) and Lymphocyte antigen 6 family member G (Ly6G, both from Wuhan Saiweier Biotechnology Co., Ltd., Wuhan, China) immunohistochemistry (IHC) with DAB visualization, and hematoxylin counterstaining. Images were captured using a slide scanner. To ensure objective and quantitative assessment, all histological slides were independently evaluated and scored by three pathologists who were blinded to the experimental group assignments.

### 2.4. qRT-PCR Analysis

Total RNA was extracted from mouse liver tissues using TRIzol reagent (Thermo Fisher Scientific, Waltham, MA, USA). Subsequently, 1 μg of total RNA was reverse-transcribed into cDNA using the PrimeScript RT Reagent Kit (TaKaRa, Kyoto, Japan) following the manufacturer’s instructions. Next, quantitative real-time PCR (qPCR) assays were performed using SYBR Green PCR Master Mix (Thermo Fisher Scientific, Waltham, MA, USA) in accordance with the manufacturer’s protocols. The primers used for qPCR are listed in Table S1. Finally, the relative expression levels of the target genes were calculated from the Ct values using the 2^−ΔΔCt^ method. *Gapdh* was used as the stable reference gene for normalization across all experimental conditions.

### 2.5. 16S rRNA Gene Sequencing

DNA was extracted from mouse cecal content. The V3–V4 hypervariable region of the 16S rRNA gene was amplified with primers 338F/806R and sequenced on the Illumina HiSeq 2500 platform (2 × 250 bp). Raw reads were quality-filtered by removing primers and adapters using Cutadapt (v2.6). Clean reads were then processed with USEARCH (v7.0.1090): sequences were clustered into Operational Taxonomic Units (OTUs) at 97% similarity using UPARSE, and chimeric sequences were detected and removed with UCHIME (v4.2.40). Taxonomic assignment was performed using the RDP classifier against the RDP database (v11.5). All samples were rarefied to an even sequencing depth before downstream analysis. α- and β-diversity (based on weighted Bray–Curtis distance) were calculated using MOTHUR and QIIME, respectively. Differences in community structure between groups were assessed with ANOSIM, and differentially abundant taxa were identified using LEfSe (LDA score > 2).

### 2.6. Statistical Analysis

All data are expressed as mean ± standard error of the mean (SEM). The normality of distribution within each group was assessed using the Shapiro–Wilk test, and homogeneity of variances between groups was evaluated with the Brown–Forsythe test. For comparisons between three or more groups, if parametric test assumptions were met, one-way ANOVA (or Welch’s ANOVA for unequal variances) was used. If not, the Kruskal–Wallis test was employed. When the overall test was significant, Dunn’s post hoc test was used for pairwise comparisons. For comparisons between two groups, the Student’s t-test or the Mann–Whitney U test was applied as appropriate. All multiple comparisons were adjusted using the two-stage Benjamini, Krieger, and Yekutieli method to control the false discovery rate (FDR). Data analysis was performed using GraphPad Prism software (v8.4.2). Statistical significance was set at * *p* < 0.05 and ** *p* < 0.01.

## 3. Results

### 3.1. The Effects of Cholic Acid and DSV on Liver Damage

**Figure 1:**
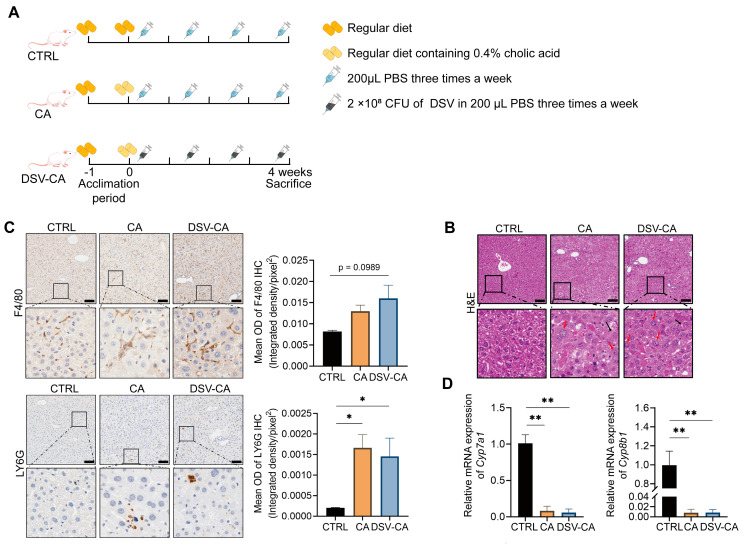
Hepatic histology and gene expressions in mice fed with the regular diet and CA diet with or without DSV administration. (**A**) Experimental design (n = 5 mice per group). (**B**) H&E staining of liver sections. The red arrows point to the enlarged nuclei. The black arrows point to the ground-glass inclusions. Representative images are shown. Histopathological assessment was performed in a blinded manner. (**C**) F4/80 and LY6G IHC staining of liver sections and quantification of mean optical density (OD) of positive areas. Scale bar: 100 μm. (**D**) Relative mRNA expression of hepatic *Cyp7a1* and *Cyp8b1* in the CTRL, CA, and DSV-CA groups. Data are represented as mean ± SEM, one-way ANOVA with FDR post hoc test (two-stage step-up method of Benjamini, Krieger and Yekutieli), * *p* < 0.05, ** *p* < 0.01.

To investigate the effects of elevated intestinal BAs concentrations on liver damage, we established a mouse model by feeding mice a 0.4% CA diet for 28 days (Figure 1A). H&E staining showed no obvious pathological damage in the CTRL group, while the CA group exhibited pathological features consistent with liver damage, including diffuse hepatocyte enlargement with blurred borders, enlarged and abnormally shaped nuclei in some cells, areas of ground-glass-like changes in the cytoplasm, and lipid vacuoles of varying sizes, suggesting significant fatty degeneration (Figure 1B). The liver pathological manifestations of the DSV-CA group were similar to those of the CA group, with no significant improvement or worsening (Figure 1B). F4/80 IHC staining showed a non-significant increase in F4/80 positive signals in the liver of mice in the DSV-CA group compared to the CTRL group (*p* = 0.0989), suggesting a potential increase in the number of macrophages (Figure 1C). Ly6G IHC staining showed that positive signals in the liver of mice in the CA group and DSV-CA group significantly increased compared to the CTRL group, suggesting an increase in the number of neutrophils (Figure 1C).

Further analysis of the expression of the key genes involved in BA synthesis showed that compared with the CTRL group, the mRNA expression of *Cyp7a1* and *Cyp8b1* was significantly decreased in the CA group (*p* < 0.01), consistent with a negative feedback inhibition of endogenous BA synthesis in response to excessive BA intake (Figure 1D). This is consistent with the well-established FXR-FGF15 axis: elevated intestinal BAs activate the farnesoid X receptor (FXR) in the ileum, leading to induction of fibroblast growth factor 15 (FGF15), which circulates to the liver and suppresses the expression of *Cyp7a1*, the rate-limiting enzyme in BA synthesis. However, DSV supplementation did not further downregulate the expression of genes in this pathway (Figure 1D). The above data demonstrate that an abnormal increase in intestinal BAs is sufficient to induce hepatic histological alterations, and that additional supplementation with DSV did not further exacerbate the histological changes observed under conditions of excessive cholic acid intake.

### 3.2. The Effects of CA and DSV on the Gut Microbiota

**Figure 2:**
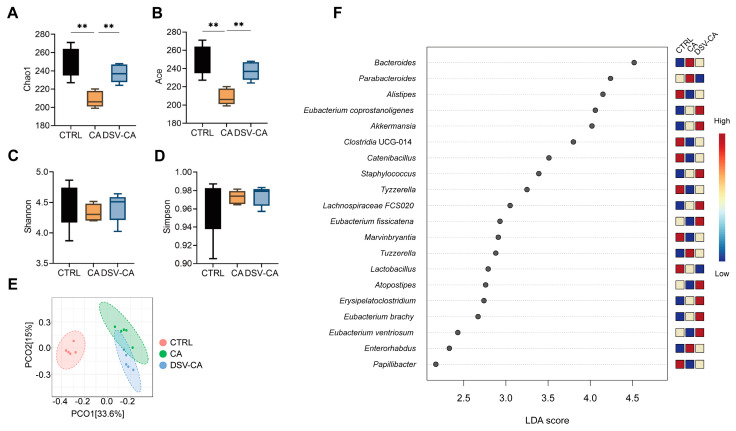
The effect of the intake of CA and DSV on the gut microbial structure in mice. α diversity index (**A**) Chao 1, (**B**) Ace, (**C**) Shannon, (**D**) Simpson. Differences between groups were analyzed by one-way ANOVA followed by the two-stage step-up method of Benjamini, Krieger, and Yekutieli for false discovery rate (FDR) correction. ** *p* < 0.01. (**E**) Beta diversity of gut microbiota of mice in the CTRL, CA, and DSV-CA groups. Principal Coordinates Analysis (PCoA) based on the weighted Bray–Curtis algorithm. (**F**) LDA score of LEfSe of mouse gut microbiota in the CTRL, CA, and DSV-CA groups. Statistical comparisons were carried out by one-way ANOVA and ANOSIM analysis, except for LEfSe analysis, which used the nonparametric Kruskal–Wallis sum-rank test and Wilcoxon rank-sum test.

To investigate the effects of CA and DSV interventions on the gut microbiota of mice, we performed 16S rRNA gene sequencing on cecal contents from mice in different treatment groups. Alpha diversity metrics assess community richness and evenness: Chao1 and Ace estimate species richness, while Shannon and Simpson indices incorporate both richness and evenness. Alpha diversity analysis revealed that the Chao1 (Figure 2A) and Ace (Figure 2B) indices in the CA group were significantly lower than in the CTRL group (*p* < 0.01), indicating that CA treatment reduced the diversity of the gut microbiota. However, the Chao1 and Ace indices in the DSV-CA group were significantly higher than in the CA group (*p* < 0.01), indicating that the DSV intervention modulated the decrease in microbial richness caused by CA, leading to a partial recovery in richness indices. The Shannon (Figure 2C) and Simpson (Figure 2D) indices showed no significant differences between the groups. Principal Coordinates Analysis (PCoA) based on the weighted Bray–Curtis algorithm showed that the CTRL group was clearly separated from the CA and DSV-CA groups along PCo1 (contributing 33.6%), indicating significant differences in community structure between the CTRL group and the two treatment groups (Figure 2E). Meanwhile, the community structures of the CA and DSV-CA groups were relatively similar (Figure 2E).

Linear discriminant analysis effect size (LEfSe) analysis identified key biomarkers with significant differences between the three groups at the genus level. Compared to the CTRL group, the CA group showed an increased abundance of genera such as *Bacteroides*, *Tuzzerella*, and *Enterorhabdus*, and a decreased abundance of genera such as *Alistipes*, *Clostridia* UCG-014, *Catenibacillus*, *Tyzzerella*, *Marvinbryantia*, and *Papillibacter* (Figure 2F). Compared to the CA group, the abundance of species such as *Eubacterium fissicatena*, *Atopostipes*, and *Eubacterium ventriosum* increased significantly in the DSV-CA group, while *Parabacteroides* abundance decreased (Figure 2F). The above results indicate the significantly altered composition of the gut microbiota. DSV intervention under high BA conditions was associated with further alterations in the microbial community, including enrichment of specific taxa such as *Eubacterium ventriosum*.

### 3.3. The Effects of Cholestyramine on Liver Damage Induced by DSV

**Figure 3:**
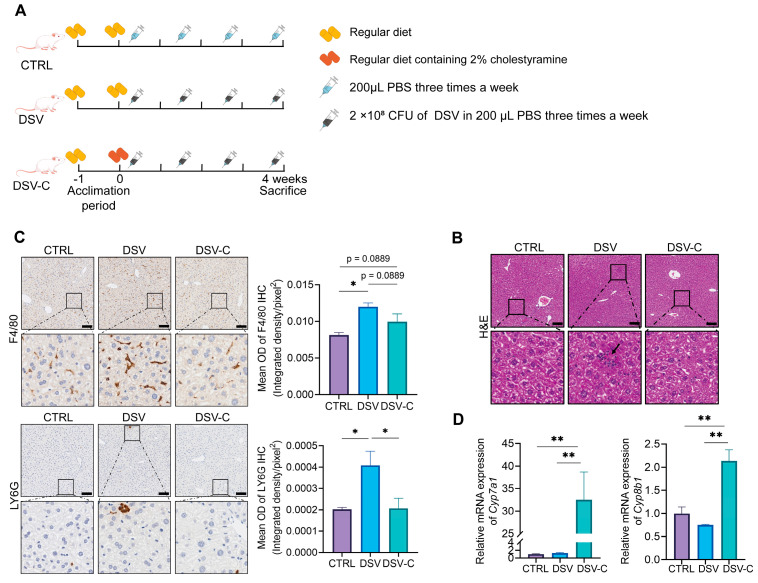
Hepatic histology and gene expressions in mice fed with the regular diet and cholestyramine diet with or without DSV administration. (**A**) Experimental design (n = 5 mice per group). (**B**) H&E staining of liver sections. The black arrow points to the inflammatory infiltration. Representative images are shown. Histopathological assessment was performed in a blinded manner. (**C**) F4/80 and LY6G IHC staining of liver sections and quantification of mean OD of positive areas. Scale bar: 100 μm. (**D**) Relative mRNA expression of hepatic *Cyp7a1* and *Cyp8b1* in the CTRL, DSV, and DSV-C group. Data are represented as mean ± SEM, one-way ANOVA with FDR post hoc test (two-stage step-up method of Benjamini, Krieger, and Yekutieli), * *p* < 0.05, ** *p* < 0.01.

To verify that DSV-induced liver injury depends on the upregulation of the intestinal BA pool, we fed mice a diet containing 2% cholestyramine, which chelates BAs and reduces their concentration in the intestine (Figure 3A). H&E staining revealed no obvious pathological abnormalities in the livers of the CTRL group. Mild focal inflammatory infiltration was occasionally observed in the livers of the DSV group. The livers of the DSV-C group were similar to those of the CTRL group, with no obvious pathological abnormalities (Figure 3B). F4/80 IHC staining showed that the F4/80 positive signal in the liver of mice in the DSV group significantly increased compared to the CTRL group (*p* < 0.05), suggesting an increase in the number of macrophages; while the F4/80 positive signal in the liver of mice in the DSV-C group showed a decreasing trend compared to the DSV group (*p* = 0.0889, Figure 3C). Ly6G IHC staining showed that the positive signal in mice in the DSV group significantly increased compared to the CTRL group (*p* < 0.05), suggesting an increase in the number of neutrophils, while the Ly6G positive signal in the liver of mice in the DSV-C group significantly decreased compared to the DSV group (Figure 3C).

The gene expression of hepatic *Cyp7a1* and *Cyp8b1* was increased in the DSV-C group compared to the DSV and CTRL groups (Figure 3D, *p* < 0.05). This transcriptional upregulation suggests a compensatory activation of the hepatic BA synthesis pathway in response to intestinal BA sequestration by cholestyramine. These results suggest that the hepatic histological alterations induced by DSV are associated with the upregulation of the intestinal BAs pool, and that cholestyramine administration can alleviate these alterations.

### 3.4. The Effects of Cholestyramine on the Gut Microbiota of Mice with Liver Damage Induced by DSV

**Figure 4:**
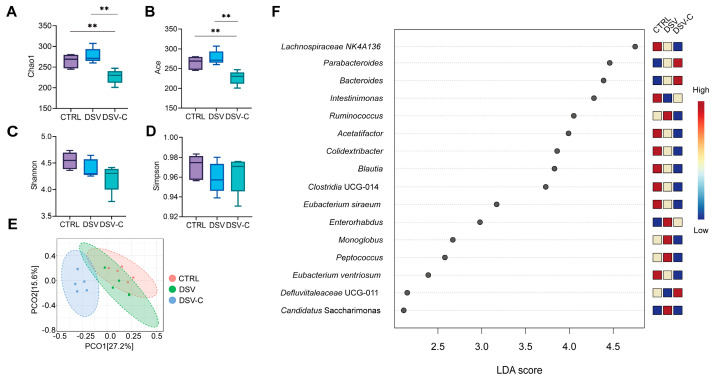
The effect of the intake of Cholestyramine and DSV on the gut microbial structure in mice. α diversity index (**A**) Chao 1, (**B**) Ace, (**C**) Shannon, (**D**) Simpson. Differences between groups were analyzed by one-way ANOVA followed by the two-stage step-up method of Benjamini, Krieger, and Yekutieli for FDR. ** *p* < 0.01. (**E**) Beta diversity of gut microbiota of mice in the CTRL, DSV, and DSV-C group. Principal Coordinates Analysis (PCoA) based on the weighted Bray–Curtis algorithm. (**F**) LDA score of LEfSe of mouse gut microbiota in the CTRL, DSV, and DSV-C groups. Statistical comparisons were carried out by one-way ANOVA and ANOSIM analysis, except for LEfSe analysis, which used the nonparametric Kruskal–Wallis sum-rank test and Wilcoxon rank-sum test.

To gain a comprehensive understanding of how cholestyramine and DSV impact the gut microbiota, we first conducted an Alpha diversity analysis (Figure 4A–D). Notably, the DSV-C group exhibited significantly lower values for both the Chao1 (Figure 4A) and Ace (Figure 4B) indices compared to the CTRL and DSV groups. This observation strongly suggests that cholestyramine has a reducing effect on the species richness of the gut microbiota. In contrast, when comparing the DSV and CTRL groups, no significant difference was detected in these two indices. The Shannon (Figure 4C) and Simpson (Figure 4D) indices showed no significant differences between the three groups. This indicates that, at least in terms of species richness as measured by these indices, DSV treatment alone does not lead to a substantial alteration compared to the control condition.

Moving on to the community structure of the gut microbiota, we performed Beta diversity analysis. The PCoA plot based on the weighted Bray–Curtis algorithm (Figure 4E) showed that the DSV-C and CTRL groups, as well as the DSV-C and DSV groups, could be clearly separated by the PCo1 axis, which accounted for a contribution rate of 27.2%. This separation implies that the treatment of cholestyramine (in the DSV—C group) leads to a significant shift in the overall community structure of the gut microbiota compared to both the control and the DSV-treated groups. On the other hand, the CTRL and DSV groups clustered together, indicating that their gut microbiota community structures were similar.

To delve deeper into the differential biomarkers among the three groups, we conducted an LEfSe analysis (Figure 4F). Compared to the CTRL group, the abundances of *Enterorhabdus* and *Candidatus Saccharimonas* were significantly higher in the DSV group, while the abundance of *Intestinimonas* was significantly lower.

In the comparison between the DSV-C and DSV groups, another set of changes emerged. The abundance of *Defluviitaleaceae* UCG-011 was significantly higher in the DSV-C group, whereas the abundances of *Ruminococcus*, *Monoglobus*, *Peptococcus*, and *Candidatus Saccharimonas* were significantly lower. This indicates that the addition of cholestyramine to the DSV treated group leads to a further reshaping of the microbial community, with specific genera being either favored or suppressed. Notably, the abundance of *Candidatus Saccharimonas*, a representative of the candidate phylum Saccharibacteria (TM7) frequently detected in host-associated environments and linked to dietary or metabolic shifts, showed a distinct pattern. It increased with DSV intervention and decreased with cholestyramine intervention. This association suggests that the abundance of *Candidatus Saccharimonas* may be linked to alterations in the intestinal BA environment. Similarly, the abundance of *Enterorhabdus* increased not only in the DSV group of this experiment but also significantly in the CA group compared to the CTRL group. This consistent increase across different experimental conditions implies changes in *Enterorhabdus* abundance are associated with conditions of altered BAs homeostasis.

## 4. Discussion

In this study, by employing complementary models of BA elevation and sequestration, we provide evidence that the BA sequestrant cholestyramine attenuated excessive DSV-triggered hepatic inflammation. This establishes the upregulation of the intestinal BA pool as an indispensable mediator in the pathogenesis of this condition.

Serving as amphipathic steroid molecules, BAs are generated in the liver from cholesterol. They play a crucial role in the emulsification of lipids, their subsequent digestion and absorption, and the uptake of fat-soluble vitamins [24,25]. However, when they accumulate to high concentrations in vivo, they exhibit cytotoxicity, induce inflammatory responses, and contribute to the pathogenesis of various liver diseases, such as alcoholic liver disease, cholestatic liver injury, and liver fibrosis [26,27,28,29]. Notably, DSV intervention further intensified the infiltration of inflammatory cells like macrophages in the CA-induced model, suggesting that DSV may exacerbate BA-related liver damage by amplifying the inflammatory response. This finding aligns with clinical observations: a positive correlation has been established between increased DSV abundance and the occurrence of multiple human diseases [30]. Notably, it is significantly enriched in patients with non-alcoholic fatty liver disease (NAFLD); furthermore, this bacterium contributes to the pathogenesis of conditions such as ulcerative colitis and colorectal cancer, potentially through mechanisms including intestinal barrier disruption [31,32,33,34,35].

Experiments involving the BA sequestrant cholestyramine provided crucial evidence that this treatment nearly completely attenuated DSV-induced hepatic inflammatory cell infiltration. This suggests that the upregulation of the intestinal BA pool may play a critical mediating role. Notably, cholestyramine intervention not only reversed DSV-associated microbial shifts but also further reduced overall species richness in the gut. This reduction in richness may be explained by a dual mechanism: first, by strongly sequestering BAs, cholestyramine removes a key ecological selective pressure that shapes community structure, potentially eliminating the growth advantage of certain bacteria that thrive in high-bile-acid conditions; second, the physicochemical properties of the resin itself may create a microenvironment selectively unfavorable to certain bacterial taxa. Thus, the decline in richness may not merely be a side effect of perturbation but could reflect a transition of the gut microbiota toward a more stable or less inflammatory state, consistent with its therapeutic effect in alleviating hepatic inflammation. As an unabsorbable anion exchange resin, cholestyramine binds to intestinal BAs to form insoluble complexes, promoting their excretion [36]. It is used clinically to regulate cholesterol levels and alleviate symptoms related to cholestasis [37,38,39]. The significant protective effect demonstrated in this study further highlights the central role of BA homeostasis in the mechanism of liver injury. Cholestyramine may potentially be used in the future for the treatment of liver diseases caused by excessive BAs resulting from intestinal dysbiosis. However, it is important to note that as a BA sequestrant, cholestyramine can also reduce the intestinal absorption of fat-soluble vitamins and other dietary lipids, which is a relevant consideration for long-term therapeutic use.

The enterohepatic circulation of BAs is central to regulating their homeostasis within the body. Around 95% of BAs are reabsorbed in the distal ileum in a conjugated form by the apical sodium-dependent BA transporter (ASBT). After returning to the liver via the portal vein, they are secreted back into the biliary tract, forming an efficient recycling system [40,41]. However, gut microbiota decouples conjugated BAs by expressing bile salt hydrolase (BSH), which inhibits their active reabsorption via ASBT and impairs the efficiency of enterohepatic circulation. BSH activity is widespread among multiple bacterial genera, including *Bacteroides*, *Clostridium*, *Lactobacillus*, and *Bifidobacterium* [42,43], establishing a critical molecular basis for the regulation of BA metabolism by the microbiota.

In addition to serving as substrates for microbial metabolism, BAs act as key regulators of gut microbial composition [29,44,45]. Their steroid backbone confers lipophilic properties, endowing them with direct antimicrobial activity. At high concentrations, they exert intense selective pressure on microbial communities by disrupting cell membrane structures, inducing protein misfolding and causing oxidative damage to nucleic acids [46]. PcoA analysis in this study revealed that the CA and DSV-CA groups had similar microbial structures but were distinct from the control group. Collectively, these results indicate that BAs serve as the primary driver of community remodeling, while DSV exerts a modulatory effect within this BAs-dominated framework, fine-tuning the microbial composition. We observed the downregulation of certain genera, such as *Lactobacillus*, under CA and DSV interventions, while *Eubacterium coprostanoligenes* and *Eubacterium fissicatena*, which are closely associated with BA metabolism, showed significant upregulation. This shift likely represents an adaptive response by the microbiota to high BA stress. Following the administration of cholestyramine to chelate intestinal BAs, *Eubacterium coprostanoligenes* and *Eubacterium fissicatena* were significantly downregulated. Previous studies have also demonstrated that a CA diet promotes the proliferation of specific bacterial groups, such as *Clostridium* [47], corroborating the findings of this study. The specific enrichment of *Eubacterium ventriosum* and *Enterorhabdus* under high cholic acid conditions suggests a microbial adaptation to metabolize excess BAs. *Eubacterium* species are key players in BA metabolism, possessing BA hydrolase activity and performing 7α-dehydroxylation to convert primary BAs into secondary ones like deoxycholic acid (DCA) and lithocholic acid (LCA)—critical signaling molecules for host homeostasis and inflammation [48,49]. Notably, *Enterorhabdus* has been directly linked to the production of DCA and LCA, with its abundance positively correlating with their levels [50]. Thus, *Eubacterium ventriosum* and *Enterorhabdus* likely form a functionally complementary metabolic alliance in response to BA stress. Their activity may increase intestinal levels of DCA and LCA, which could then modulate host BA synthesis and inflammation via receptors like FXR in the gut–liver axis, contributing to DSV-induced liver injury.

This study confirms that the effects of DSV depend on disruption of the BA pool, and the underlying mechanisms may be closely related to the regulation of the gut microbiota–BAs–FXR axis. We speculate that DSV may interfere with the host FXR signaling pathway through its characteristic metabolite H_2_S, thereby affecting BA synthesis and cholesterol metabolism. H_2_S produced by DSV can induce hepatic FXR signaling, inhibit CYP7A1 expression, and promote cholesterol secretion, thereby driving biliary cholesterol supersaturation [22]; another study also suggested that modulating the gut microbiota can inversely affect FXR signaling activity and BA metabolism [51]. The observed changes in hepatic *Cyp7a1* expression in our study align with the direction of the above mechanisms. Future research should further dissect this regulatory network at metabolic and transcriptional levels.

This study has several limitations that should be acknowledged. First, the experimental design lacked a separate cholestyramine-only control group. Given that the resin may exert non-specific effects on intestinal physiology, the observed improvements cannot be fully attributed to BA sequestration. Second, the assessment of liver injury relied on histopathology without the measurement of serum biochemical markers such as ALT and AST. This limits the ability to functionally and quantitatively evaluate the severity of hepatocellular damage. Third, the absence of targeted BA metabolomics data hinders a deeper mechanistic interpretation, as we were unable to identify the specific BA species altered by DSV or to quantify changes in the enterohepatic pool. Such information is essential to distinguish whether the observed effects result from altered synthesis, bacterial transformation, or absorption. Fourth, several limitations pertain to the microbial analyses. (1) Critically, the administered DSV strain did not establish stable colonization, meaning the observed effects are attributed to repeated exposure rather than chronic colonization. (2) Consequently, the 16S rRNA data, while revealing community shifts, cannot establish causality between specific bacterial changes and the phenotype. (3) Furthermore, 16S rRNA gene sequencing provides only taxonomic information; it cannot identify the functional genes responsible for the observed microbial shifts in BAs metabolism. Future functional metagenomics studies are needed to directly link microbial community changes to functional genetic potential. (4) The functional roles of highlighted bacteria remain speculative. Together, these points refine our model to one of acute bacterial challenge triggering BA-mediated injury, a mechanism that future studies with colonizing strains or defined metabolites can test directly. Fifth, the sample size per group (n = 5), while common in preliminary studies, may limit the statistical power to detect subtle effects. Finally, the doses and timing of interventions in this study were based on experimental standards. Their clinical translational potential requires systematic investigation, including dose–response relationships, optimal therapeutic windows, and potential synergistic effects with microbiota-targeted therapies. Additionally, the use of only male mice limits the exploration of potential sex-dependent differences in the gut microbiota–bile acid–liver axis, which should be considered in future research.

Addressing these limitations in future research will help deepen the understanding of the role of bile acid modulation in gut microbiota-associated liver injury and enhance its translational potential.

## Data Availability

The original contributions presented in this study are included in the article/Supplementary Material. Further inquiries can be directed to the corresponding authors.

## Supplementary Materials

The following supporting information can be downloaded at https://www.mdpi.com/article/10.3390/microorganisms14010079/s1, Table S1: Primers for quantitative real-time PCR.
