# Supplementary material for: Bile Acid Sequestration Attenuates Desulfovibrio-Induced Hepatic Injury
Source: Microorganisms. 2025 Dec 30;14(1):79. doi: 10.3390/microorganisms14010079 (PMC12843977; doi:10.3390/microorganisms14010079)
Supplement: Supplementary file 1 [file microorganisms-14-00079-s001.zip › microorganisms-4009815-supplementary.pdf]

Supplemental Table

Table S1 Primers for quantitative real-time PCR

Gene	Sequence of forward primers (5' to 3')	Sequence of reverse primers (5' to 3')
Gapdh	AGGTCGGTGTGAACGGATTTG	TGTAGACCATGTAGTTGAGGTCA
Cyp7a1	AGCAACTAAACAACCTGCCAGTACTA	GTCCGGATATTCAAGGATGCA
Cyp8b1	CATGAAGGCTGTGCGTGAGGAA	CATCACGCTGTCCAACACTGGA
